# CO_2_ supply is a powerful tool to control homoacetogenesis, chain elongation and solventogenesis in ethanol and carboxylate fed reactor microbiomes

**DOI:** 10.3389/fbioe.2024.1329288

**Published:** 2024-04-24

**Authors:** Kasper D. de Leeuw, Marius J. W. van Willigen, Ton Vrauwdeunt, David P. P. T. B. Strik

**Affiliations:** ^1^ Environmental Technology, Wageningen University and Research, Wageningen, Netherlands; ^2^ ChainCraft B.V., Amsterdam, Netherlands

**Keywords:** chain elongation, CO_2_, ethanol, acetogenesis, solventogenesis, reduction, carboxylates, hexanol

## Abstract

Anaerobic fermentation technology enables the production of medium chain carboxylates and alcohols through microbial chain elongation. This involves steering reactor microbiomes to yield desired products, with CO_2_ supply playing a crucial role in controlling ethanol-based chain elongation and facilitating various bioprocesses simultaneously. In the absence of CO_2_ supply (Phase I), chain elongation predominantly led to n-caproate with a high selectivity of 96 Cmol%, albeit leaving approximately 80% of ethanol unconverted. During this phase, *C. kluyveri* and *Proteiniphilum*-related species dominated the reactors. In Phase II, with low CO_2_ input (2.0 NmL L^−1^ min^−1^), formation of n-butyrate, butanol, and hexanol was stimulated. Increasing CO_2_ doses in Phase III (6 NmL L^−1^ min^−1^) led to CO_2_ utilization via homoacetogenesis, coinciding with the enrichment of *Clostridium luticellarii*, a bacterium that can use CO_2_ as an electron acceptor. Lowering CO_2_ dose to 0.5 NmL L^−1^ min^−1^ led to a shift in microbiome composition, diminishing the dominance of *C. luticellarii* while increasing *C. kluyveri* abundance. Additionally, other *Clostridia*, *Proteiniphilum*, and *Lactobacillus sakei*-related species became prevalent. This decrease in CO_2_ load from 6 to 0.5 NmL L^−1^ min^−1^ minimized excessive ethanol oxidation from 30%–50% to 0%–3%, restoring a microbiome favoring net n-butyrate consumption and n-caproate production. The decreased ethanol oxidation coincided with the resurgence of hydrogen formation at partial pressures above 1%. High concentrations of butyrate, caproate, and ethanol in the reactor, along with low acetate concentration, promoted the formation of butanol and hexanol. It is evident that CO_2_ supply is indispensable for controlling chain elongation in an open culture and it can be harnessed to stimulate higher alcohol formation or induce CO_2_ utilization as an electron acceptor.

## Introduction

Transitioning from fossil feedstocks to renewables in the production of bulk and fine chemicals is essential to meet the needs of future generations (Lange, 2021). To evolve from a petrochemical industry into a circular and sustainable one, it is crucial to explore and harness alternative carbon sources like residual biomass streams, household wastes, and various CO_2_ streams (Sherwood, 2020). The conversion of these resources into valuable chemicals can be achieved through diverse (electro) (bio) refinery approaches (Harnisch and Urban, 2018; Takkellapati et al., 2018; Rehan et al., 2019; Platt et al., 2021). The Carboxylate platform, also known as the VFA (volatile fatty acids) platform, represents a promising opportunity in this transformation (Kim N et al., 2018; Holtzapple et al., 2022). At the core of this platform are bioreactors where microorganisms produce a spectrum of carboxylates. Various industries are currently successfully commercializing VFA value chains, converting biomass residues into, for instance, animal feed additives (Strik et al., 2022).

Within the carboxylate platform, microbial chain elongation bioprocesses are gaining increased attention, offering a broader product spectrum with the formation of medium-chain carboxylates (MCCA) and their corresponding alcohols (Angenent et al., 2016; Wu et al., 2020). These bioprocesses are executed by undefined mixed cultures, commonly referred to as reactor microbiomes, or through defined pure or co-cultures utilizing various carbon-chain elongation pathways like homoacetogenesis and reverse β-oxidation (Spirito et al., 2014). Effective processes have been developed that leverage short carbon chain carboxylates, derived from organic municipal solid wastes, and elongate them through the supply of ethanol as an electron donor, resulting in the production of medium-chain carboxylates (Grootscholten et al., 2014; Roghair et al., 2018a). *Clostridium kluyveri*, a well-studied microorganism for ethanol-based chain elongation, utilizes acetate with ethanol to produce butyrate, caproate, and H_2_ (Seedorf et al., 2008). Additional chain elongation bacteria include strains like *Megasphaera elsdenii, Megaspheara hexanoica, Pseudoramibacter alactolyticus, Ruminococcaceae bacterium CPB6,* and *C. luticellarii* (Kim H et al., 2018; Liu et al., 2020; Candry and Ganigué, 2021). Certain strains, such as *C. luticellarii*, exhibit the capability to perform chain elongation from CO_2_ up to (iso) butyrate and caproate (K. D. de Leeuw et al., 2020; Petrognani et al., 2020).

Solventogenesis is commonly observed in microbial chain elongation bioprocess development, leading to the production of not only carboxylates but also alcohols beyond ethanol, including branched alcohols (de Leeuw et al., 2021; Robles et al., 2023). Various pathways are now considered for alcohol formation in the development of chain elongation bioprocesses, including: 1) hydrogenotrophic carboxylate reduction (e.g., butyrate reduction to butanol with hydrogen) (Steinbusch et al., 2008): 2) carboxyl-hydroxyl exchanging, which couples hydrogenogenic ethanol oxidation with hydrogenotrophic carboxylate reduction (K. D. De Leeuw et al., 2021)); 3) carbon monoxide-driven carboxylate reduction (Diender et al., 2016; Richter et al., 2016); 4) bioelectrochemical carboxylate reduction using electrons or hydrogen from a cathode (Sharma et al., 2013); or 5) alcohol production as an apparent result of the ethanol-based chain elongation process itself, such as propanol, butanol, and hexanol, as demonstrated with strains from *Clostridium* kluyveri (Kenealy and Waselefsky, 1985; Candry et al., 2020).

The steering of open culture chain elongation processes involves the meticulous control of various parameters, including pH, temperature, substrate (electron donor and acceptor) species and concentrations, N_2_ and CO_2_ gas supply, H_2_ partial pressure, and hydraulic retention time (HRT) (Contreras-Dávila et al., 2021a; Contreras-Dávila et al., 2021b; De Groof et al., 2020; K. D; de Leeuw et al., 2020; Grootscholten et al., 2013; Robles et al., 2023; Roghair, Hoogstad, et al., 2018a; Shrestha et al., 2023). CO_2_ gas supply stands out as a particularly crucial parameter, given its dual role as growth nutrient for chain elongating organisms and as an electron acceptor for various other microbes (Tomlinson, 1954; Tomlinson and Barker, 1954). This sets the stage for a competition between ethanol-based chain elongators, dependent on CO_2_ for their anabolism, and other microbes capable of catabolic CO_2_ reduction.

During ethanol-based chain elongation in open cultures, CO_2_ supply affects ethanol utilisation. Especially at relatively high CO_2_ loads (at 2.5 L CO_2_/L per day) a resulting higher excessive ethanol oxidation (EEO) leads to an increase of costly ethanol and base use (Roghair, Hoogstad, et al., 2018a). The EEO bioprocess is attributed to ethanol-oxidizing microorganisms that do not engage in chain elongation. The hydrogen released during ethanol oxidation can be used by synthophic partners such as hydrogenotrophic methanogens that use part of the CO_2_ as electron acceptor to produce methane while keeping H_2_ concentrations low (Roghair et al., 2018b). Alternatively, CO_2_ could also be used for homoacetogenesis, in which H_2_ is utilized to produce acetate and other biochemicals (Müller, 2019); some acetogens have shown the capability to produce butanol and hexanol (Thunuguntla et al., 2024).

In this study, we explored the feasibility of leveraging CO_2_ supply in an open-culture ethanol-based chain elongation system to control CO_2_ fixation and higher alcohol production. Previous research demonstrated that a high ethanol to acetate ratio, combined with a high carboxylate to corresponding alcohol ratio establishes a thermodynamic driving force for carboxylate reduction coupled to ethanol oxidation (K. D. De Leeuw et al., 2021). In this research we tested the hypothesis that enrichment of acetogens with CO_2_ is necessary to stimulate the higher alcohol formation, given their recognized ability to produce such alcohols. It was anticipated that once such a culture is established, decreasing CO_2_ supply would lead to a stable system where carboxylates from the chain elongation microbiome are consistently reduced to their corresponding alcohols. Additionally, the study assesses the competition between chain elongators utilizing carboxylates as electron acceptors and other microbes utilizing CO_2_ as an electron acceptor.

## Materials and methods

The research was carried out with two benchtop open culture bioreactors with carrier material (retentostat). The medium contained relatively high amounts of butyrate and ethanol to stimulate the formation of caproate. Moreover, acetate was fed in low amounts to create acetate limited conditions. The aim was to impose a thermodynamic driving force to stimulate carboxylate reduction coupled to ethanol oxidation.

### Retentostat setup

The continuous experiments were carried out in two independent 2-L jacketed continuous retentostats controlled using an ADI 1010 Bio Controller and Power Unit (Applikon, Schiedam, Netherlands) as reported before (Contreras-Dávila et al., 2021c). Both retentostats have an internal diameter and height of 105 and 240 mm, respectively. A porous polyester fabric was attached around the inner circumference of the reactor and had a thickness of 8 mm and a height of 90 mm. The fabric functioned as carrier material to stimulate biofilm formation and retain biomass. The working volume of the reactor, including the volume of the carrier material, was 1 L (Supplementary Figures S9, S10). The bioreactor was operated at a constant temperature of 35°C, a pH of 6.50 (controlled using 1.0 M until day 55, from then on 2.0 M KOH was used to lower the effect of base dosage on HRT) and a mixing speed of 100 RPM. Mixing was provided by two flat-blade disc turbines and a propeller attached to a motor. Additionally, three baffles were used to improve mixing.

Continuous operation was achieved by pumping medium in the system at a (measured by influent inflow) flowrate of 522 ± 22 mL day^−1^ for Reactor 1 and 498 ± 32 mL day^−1^ for Reactor 2. Base dosage was automated, and in effect caused HRT to also be dependent on pH control requirements (See Supplementary Figures S6, S8 for the daily base consumption). This lead to significant variations in HRT (Supplementary Figures S5, S7; Supplementary Table S1), especially during phase II (in reactor 1) and phase III (in both reactors), when CO_2_ utilization had gained momentum and additional lye dosage was required due to acid formation. Upon lowering the CO_2_ dosage in phase IV, this phenomenon was less prevalent and the HRT was maintained stably at 42.8 ± 1.0 h and 42.4 ± 0.7 h for reactor 1 and 2, respectively. In effect, the two reactors that were set up to operate under similar conditions, only did so during phase IV. The solid retention time (SRT) was not controlled, and biomass was allowed to accumulate on the porous polyester fabric carrier material.

### Medium

The retentostats were fed with 600 mmol Carbon L^−1^ (mMC) butyrate, 50 mMC acetate, 1200 mMC ethanol, and 1 g L^−1^ yeast extract as carbon sources. Butyrate was added to promote caproate formation and the Cmolar ratio of acetate to ethanol was set to 1:24 to promote longer chain alcohol formation. The same macro- and micronutrient formulations and dosages as described in (K. D. De Leeuw et al., 2021) were used (g L^−1^): NH_4_H_2_PO_4_ 3.60; MgCl_2_·6H_2_O 0.33; MgSO_4_·7H_2_O 0.20; CaCl_2_·2H_2_O 0.20; KCl 0.15. The micronutrients (Pfennig trace metals and B-vitamins) were formulated according to (Phillips et al., 1993).

### Different CO_2_ dosage regimes

The experiment can be divided into four phases of different CO_2_ loading rates. A summary of the operating conditions is shown in Supplementary Table S1. An L-type sparger was used for the addition of CO_2_ or N_2_, which was supplied using a 50 mL/min and 40 mL/min mass flow controller (Bronkhorst, Veenendaal, Netherlands), respectively. Gas leaving the reactor passed through a volumetric gas flow meter (BCP Instruments, Lund, Sweden).

During the first phase, no CO_2_ gas was dosed. During the second phase, 2.0 NmL CO_2_/L_reactor_/min (equals 2.8 L CO_2_.L^−1^_reactor_.d^−1^) was dosed. In the third phase of the experiment, the CO_2_ inflow was further increased to 6.0 NmL/min. To prevent the development of under pressure (due to CO_2_ consumption) in the headspace of the reactors, N_2_ was also dosed at 6.0 Nml/min from this point on. Finally, during the fourth phase, the CO_2_ inflow was lowered to 0.5 NmL/min.

### Inoculum

The retentostats were inoculated using an undefined anaerobic culture mixture taken from two Upflow Anaerobic Sludge Blanket reactors that elongated acetate and isobutyrate (i-C_4_) with ethanol to (branched) MCFAs and promoted their subsequent conversion to their corresponding longer chain alcohols (K. D. De Leeuw et al., 2021). Equal volumes from both reactors were mixed (±100 mL total). Subsequently, the inoculum was decanted to remove some particulate matter. Of the remaining suspension 2 × ± 33 mL was centrifuged and the liquid phase was discarded. The pellet was washed and was subsequently resuspended in medium before being used to inoculate both retentostats. All vessels and tubes containing the inoculum were sparged using nitrogen to ensure anaerobic conditions.

### Sampling and measurements

Reactor check-ups were performed three times a week, during which both the headspace and reactor medium were sampled. The gas flow, redox values, pH, temperature, feed bag and base weight were noted during each reactor check-up. From the headspace, 2.0 mL was taken for O_2_, N_2_, CH_4_, and CO_2_ quantification using gas chromatography (Shimadzu GC-2010, Japan, in parallel with a combination of Porabond Q and Molsieve 5A, µ-TCD) and 100 µL for H_2_ measurements (HP 6890 GC, United States, Molsieve 5A, µ-TCD). Quantification was performed using established protocols for gas chromatography (Steinbusch et al., 2011; Chen et al., 2016). At the same time, ±1.8 mL medium was sampled in duplo for fatty acid and alcohol quantification. The liquid samples were centrifuged at 10,000 RFC for 10 min and stored in a freezer at −20°C (Jourdin et al., 2019). The samples were analysed within 2 weeks using gas chromatography (Agilent 7890B, United States, HP-FFAP column, FID detector) based on an earlier established protocol (Jourdin et al., 2019). The compounds of interest were primary alcohols (C_2_–C_6_) and volatile fatty acids (C_2_–C_8_). Branched volatile fatty acids were also quantified, although these were not of main interest.

### Microbial community analysis

Throughout the experiment, biomass samples of both the reactor medium and the biofilm were taken. This was done at the end of each phase, except for phase II. Biofilm biomass samples were taken by inserting a syringe throughout a port in the headplate of the reactor whilst sparging the reactor headspace with N_2_ at 50 NmL min^−1^. A plastic tube was added to the syringe to ensure it reached the carrier material. This way, a local biomass sample from the carrier material could be taken. The end of the plastic tube was placed to the carrier material after which ±10 mL of biomass sample was taken from the reactor. The samples were stored in an Eppendorf tube, centrifuged at 10.000 RFC for 10 min and the supernatant discarded. The remaining biomass pellet was frozen using liquid N_2_ and stored in the freezer at −80°C until 16s rRNA analysis was performed.

DNA extraction and 16s rRNA analysis were performed as described in De Smit et al. (2019). Subsequent taxonomic analysis was performed using the Seaborn, Matplotlib and Pandas Python packages (Waskom, 2021; Caswell et al., 2023; Pandas development team, 2023). The unconstrained redundancy triplot was generated using Canoco5.0 (ter Braak and Smilauer, 2012). The presented results are the averages of in duplo analyses.

### Steady state characterization and excessive ethanol oxidation calculations

Steady state definition and EEO were determined as described by (K. D. De Leeuw et al., 2021). Following this procedure, it was determined that only phase IV could be described as being in steady state.

### Selectivity

The C-mol selectivity for product formation was calculated by dividing the C-molar total amount of the respective product in the effluent by the sum total Carbon of all formed products. The C-mol production selectivity is through the document referred to with Cmol%. The averaged carbon and electron balances throughout the experiment are depicted in Supplementary Table S1.

**FIGURE 1:**
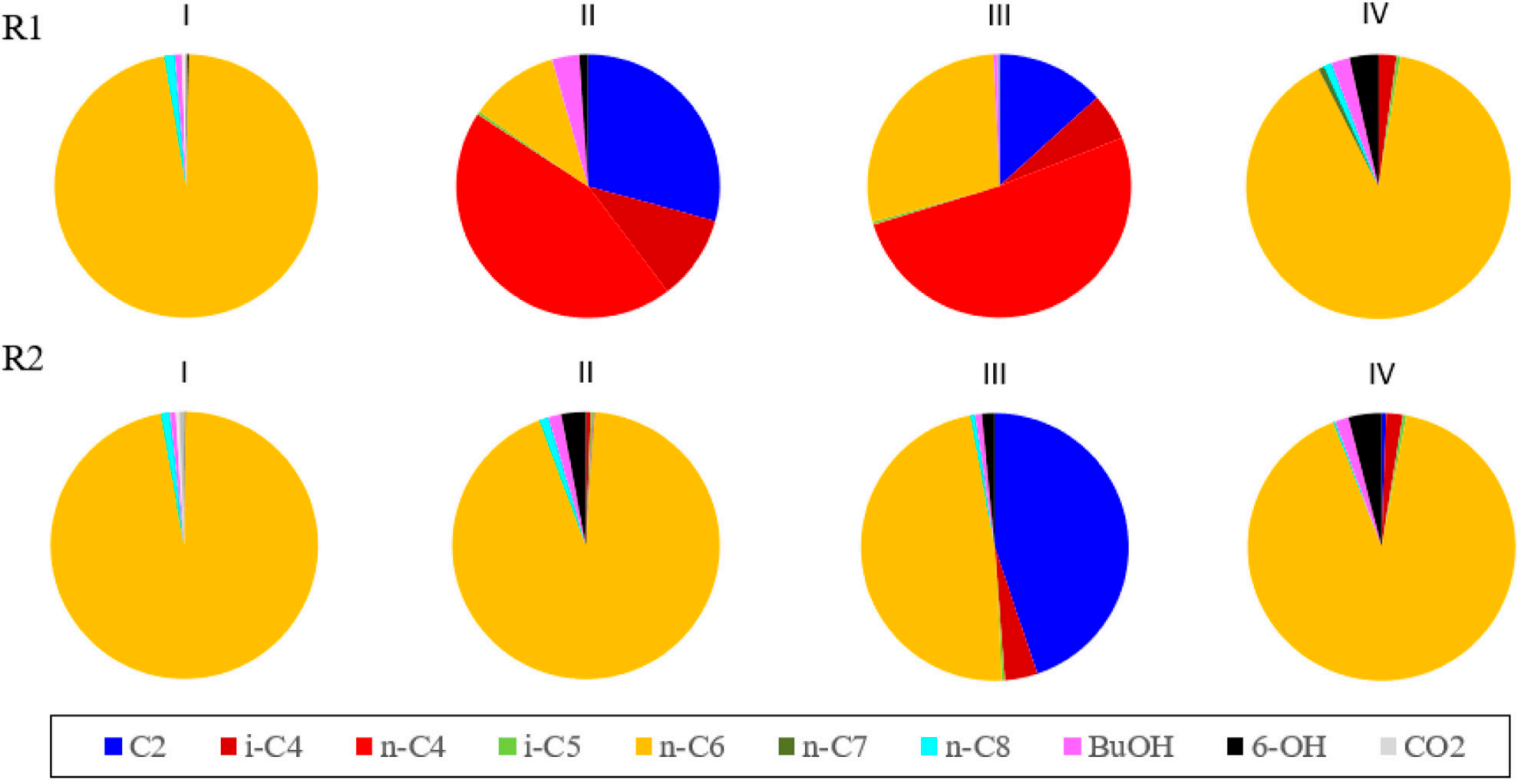
The figure depicts the average product Cmol% selectivity for reactor 1 (R1) and reactor 2 (R2) at the end of each phase. The values were calculated from the data points just before continuing to the next phase (Sample size and numerical data is given in TABLE S.2 and TABLE S.3 in the Supplementary Material). Important to note here that the pie charts only reflect the percentile carbon distribution among the formed products, no information is embedded about the absolute amounts or consumption of substrates.

When making Figure 1 and constructing the unconstrained redundancy triplot, reactor performance averages of the data points close to the preceding the start of the new phase were used. Sample size and actual period is given in Supplementary Tables S2, S3. This was done to describe the reactor activity and relate this to the microbial composition at that point in time.

## Results and discussion

The two retentostats were operated independently from each other and developed a different activity especially in phase II and III when CO_2_ dosage was ramped up. In phase IV the performance of both reactor converged to a similar profile again. For both reactors no clear steady state was established during phase I. Also during phase II and phase III no steady state was established in both reactors due to the slow and accumulating ingrowth of acetogenic activity and higher alcohol production. The only semblance of a steady state for both reactor was during phase IV, before the accidental batch phase on day 103 and during the final week of the experiment day 124 to day 133.

The reactor performance development did not occur synchronously in both reactors. Reactor 1 developed CO_2_ fixation already at the end of phase II, whereas Reactor 2 started significant CO_2_ fixation later on in phase III. Due to a sudden increase in CO_2_ consumption and resulting headspace underpressure in reactor 1 (causing troubles with the water lock), it was decided to add Nitrogen gas in the ingoing gas mix and to continue phase III. During phase III the even higher CO_2_ loading rate led to a further increase of ethanol oxidizing and CO_2_ reducing activity. With the goal to achieve a stable higher alcohol production, the authors chose to continue with phase IV, eventually leading to lowering CO_2_ a gain to maintain the system in a stable.

**FIGURE 2:**
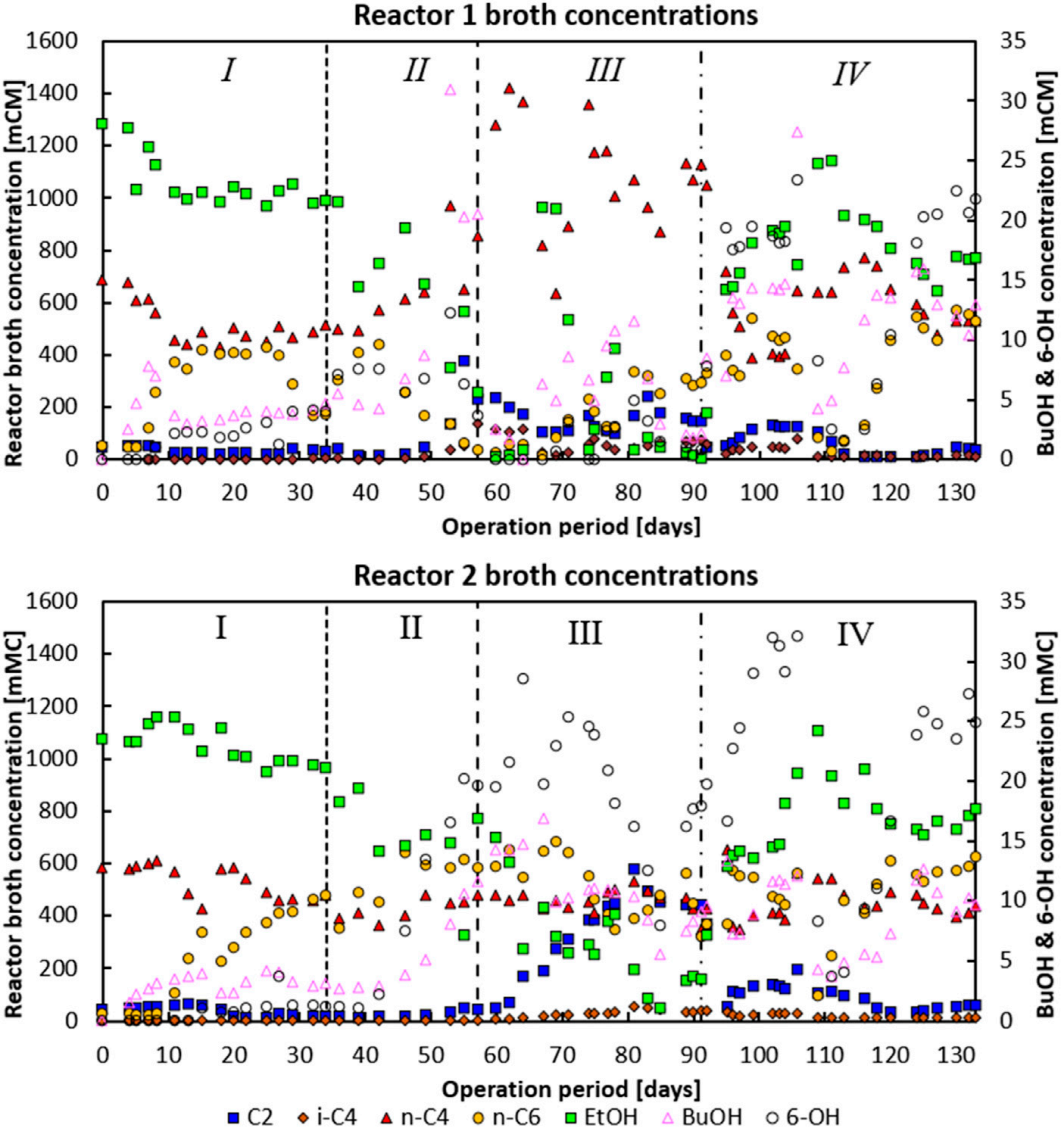
Phase I: Broth concentrations of the main metabolites for reactor 1 (top) and reactor 2 (bottom). Both reactors were in batch mode from days 0–5. Moreover, for reactor 2 the pH feed pump malfunctioned during days 11–15, leading to a temporarily lower feed dosage rate. Phase II: An under pressure had developed on day 53 for reactor 1 and on day 57 for reactor 2, respectively. Phase IV: On day 104–106 a temporary batch mode lasting 2 days occurred where no substrate was fed and no base was added, allowing pH in both systems to drop to −6.38.

Both reactors showed ethanol-based chain elongation of acetate and butyrate to caproate in varying degrees. During the whole experiment, no methane was detected. The reactor broth concentrations of the main metabolites are depicted in Figure 2. Carbon and electron balances of both reactors are shown in Supplementary Figures S1–S4. The end-of-phase averaged performances are given in Supplementary Tables S2, S3.

### CO_2_ “overloading” leads to CO_2_ utilization and can disrupt chain elongation

In the first phase, when no CO_2_ was dosed at all, both reactors consumed n-C_4_ and produced n-caproate (n-C_6_) as the main product with 97 Cmol% selectivity for both reactors near the end of the phase (see data in Figure 1). Around 80% of the fed ethanol remained unconverted, while the acetate concentration was consistently below 10 mM (0.6 g L^−1^) in the second part of this phase. In both reactors a net n-butyrate consumption was observed; n-butyrate was taken up as electron acceptor during chain elongation towards n-C_6_.The hydrogen partial pressures in both reactors during this period were around −80 kPa. Both reactors showed only little butanol and hexanol formation during this period. In this first phase in both reactors small bubbles were observed on the carrier material upon which biofilm grew. This was likely the H_2_ produced due to chain elongation as part of the reversed-beta oxidation pathway (Seedorf et al., 2008) (see Supplementary Figure S1).

In the second phase, on day 34, the CO_2_ dosage was set to 2.0 mL L^−1^ min^−1^. At the end of phase two, homoacetogenic and ethanol oxidation activity seemingly picked up in reactor 1, indicated by an increase of CO_2_ consumption from −5.7 mmol CO_2_ L^−1^ day^−1^ on day 46 to −115 mmol CO_2_ L^−1^ day^−1^ at the end of phase II (day 57). This increase in CO_2_ consumption correlated with a switch from acetate consumption towards acetate production with conversion rates going from −16.1 mCmol L^−1^ day^−1^ to 119 mCmol L^−1^ day^−1^ on days 46 and 57, respectively. The onset of acetate formation together with ethanol and CO_2_ consumption suggests the enrichment of a combined ethanol oxidative and homoacetogenic activity (Bertsch and Müller, 2015). Concomitant with this change a significant decline in n-C_6_ production and a switch from n-C_4_ consumption to n-C_4_ production (together with a large spike in butanol formation) was observed (see days 46–57). Besides, the CO_2_ utilisation reached such levels that pH_2_ dropped to below 1 kPa, while the aforementioned underpressure developed in the headspace of the reactor.

Reactor 2 did not exhibit this behaviour; it did show a significant increase in butanol and hexanol production over phase II, increasing from 1.4 to 0.6 mCmol L^−1^ day^−1^ (day 36), respectively, up to 6.48 and 11.09 mCmol L^−1^ day^−1^, respectively (day 57). Interestingly, the alcohol formation steadily increased once CO_2_ dosage was initiated and not when solely chain elongation activity was present (in phase I). This suggests that CO_2_-utilizing bacteria, e.g., acetogens, are largely responsible for the carboxylate reduction towards alcohols, and not the chain elongating bacteria (Lee et al., 2019). In reactor 1 CO_2_ was consumed together with H_2_ (>1 kPa at the end of phase II), while reactor 2 still had large amounts of CO_2_ and H_2_ (10 kPa at the end of phase II) available in the headspace.

On day 57 CO_2_ loading rates of both reactors (phase III) were adjusted to 6 Nml L^−1^ min^−^. Reactor 1 had shifted towards production of mainly n-C_4_, while n-C_6_ and hexanol production was relatively low compared to the other phases. In contrast, reactor 2 maintained n-C_6_ production, in combination with a large acetate productivity. In this system the hexanol production spiked until the ethanol concentration in the reactor dropped around day 80; chain elongation to n-C_6_ then also slightly decreased, while acetate formation spiked.

The dependency of carboxylate reduction coupled to ethanol oxidation in a proposed carboxyl-hydroxyl exchange reaction has been described in earlier research (de Leeuw et al., 2021; Robles et al., 2023). The drop in chain elongation activity with coinciding acetate formation shows that substrate competition between chain elongators and acetogens took place with the introduction of high amounts of CO_2_ as an additional electron acceptor (Katsyv and Müller, 2020; Candry and Ganigué, 2021). Interestingly, i-C_4_ formation also occurred during the spike in n-C_4_ formation (in reactor 1) and acetate formation (in reactor 2) up to a concentration of −136 mCM and −54 mCM, respectively.

### CO_2_ dosing initiated longer chain alcohol formation, but lowering CO_2_ dosage eventually maintained it

Phase IV was initiated on day 91 when the CO_2_ loading rate was lowered from 6 Nml L^−1^ min^−1^ to 0.5 Nml L^−1^ min^−1^. This significantly halted n-butyrate formation in reactor 1 (from 305.8 mCmol L^−1^ day^−1^ to −27.3 mCmol L^−1^ day^−1^) and reduced acetate formation in reactor 2 (from 86.1 mCmol L^−1^ day^−1^ to 13.9 mCmol L^−1^ day^−1^). Both systems developed a comparable performance with predominant n-C_6_ chain elongation (299 mCmol L^−1^ day^−1^ and 328 mCmol L^−1^ day^−1^ for reactor 1 and 2, respectively) and a maintained butanol and hexanol production of respectively 7.6 and 11.7 mCmol L^−1^ day^−1^ (141 mg butanol L^−1^.d^−1^ and 267 mg hexanol L^−1^.d^−1^) for reactor 1, and 14.4 and 15.9 mCmol L^−1^ day^−1^ (199 mg butanol L^−1^.d^−1^ and 271 mg hexanol L^−1^.d^−1)^ for reactor 2.

The bioreactor performance in phase IV was robust, in the sense that the selection pressure was selective enough to have the system recover to a similar performance profile after a big disturbance: on day 104, the feed pump was off for a total of 2 days, and the base pump was off for 1 day. This initiated a temporary batch phase with acidification to pH 6.37.

A large drop in activity occurred with a subsequent recovery in the following weeks. In this last phase, the hydrogen partial pressure (that had dropped to levels below 0.1 kPa in phase III) rose again to above 1 kPa. This coincided with the resurgent observation of small bubbles being produced at the biofilm on the carrier material (see Supplementary Figure S1) as also observed during phase I. A crash in acetate productivity co-occurred with the resurgence of bubbles. The progression of the CO_2_ and hydrogen partial pressure is shown in Supplementary Figure S11. The operating conditions in this phase led to very strongly similar reactor performances, whereas previously the performances large diverged. These diverging and subsequently converging reactor performances are reflected in the averaged product Cmol% selectivities at the end of each phase as depicted in Figure 1.

Longer chain alcohol formation decreased dramatically in reactor 1 at the start of phase III when ethanol was completely consumed, and the reactor produced high amounts of n-C4. Similarly, alcohol formation dropped in reactor 2 around day 85 when ethanol was almost depleted. Longer chain alcohol productivities did not recover or stabilise in reactor 1 until the start of phase IV when the CO_2_ loading rate was lowered to 0.5 Nml L^−1^ min^−1^. Ultimately the CO_2_ dosage approach led to a two similar bioreactors wherein the average longer chain alcohol selectivity rose from 1.2 Cmol% (in phase I) to 5.8 Cmol% (in phase IV). In reactor 2, also in stage IV, eventually the highest hexanol productivity of 0.3 g L^−1^ day^−1^ (17.9 mCmol L^−1^ day^−1^) and maximum concentrations of 218 mg L-1 butanol and 605 mg L-1 hexanol were achieved.

### Excessive ethanol oxidation and CO_2_ reduction towards acetate

**FIGURE 3:**
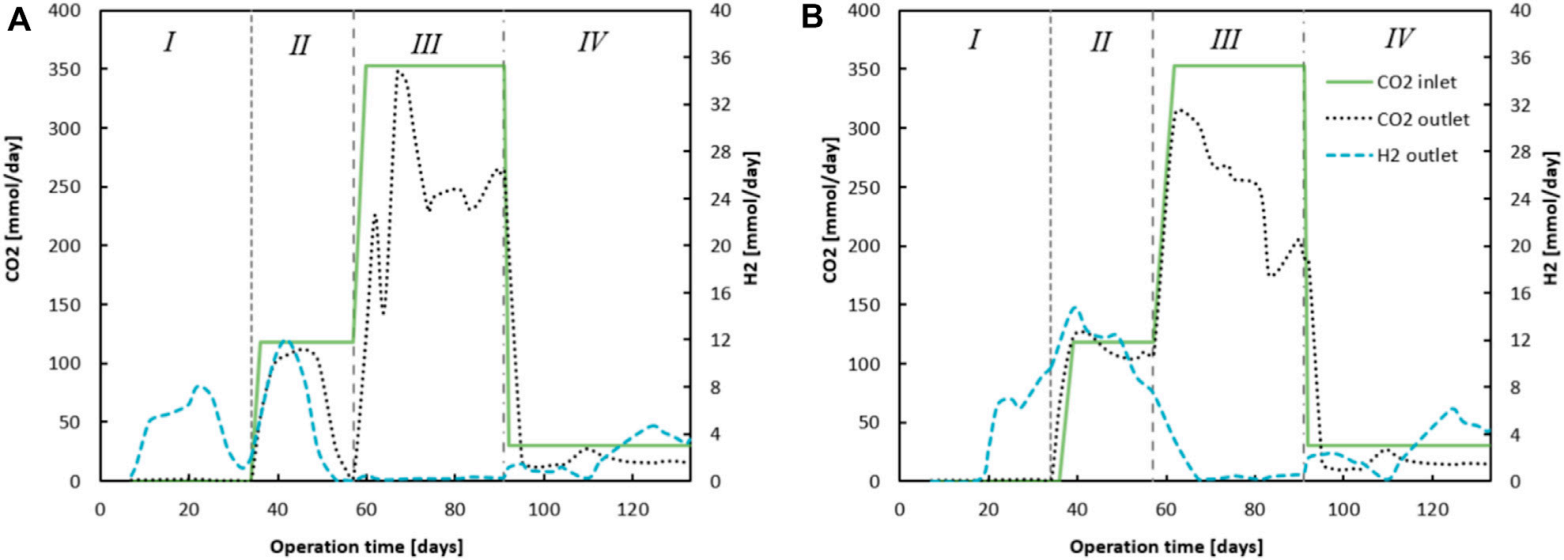
The figures show the difference between ingoing and outgoing CO_2_ (summed dissolved in effluent - and gaseous CO_2_). On the secondary *y*-axis the outgoing H_2_ gas for reactor 1 **(A)** and reactor 2 **(B)** is plotted, calculated to mmols H_2_ via the measured volumetric output and headspace composition analyses.

The progression of ingoing and outgoing CO_2_ is shown in Figure 3; the difference between these numbers indicates CO_2_ utilisation. The consumption of CO_2_ was most predominant in phase III when CO_2_ supply was highest. Supplementary Tables S2, S3 in the show the averaged values of the performance parameters (conversion rates, concentrations and EEO) for both reactors during the different phases. The CO_2_ loading rate influenced the extent to which EEO took place, similar to how earlier described (Roghair et al., 2018b). However, no methanogenesis occurred in this system. Instead, homoacetogenic activity together with carboxylate reduction towards alcohols were the main contributors towards H_2_ (from Chain Elongation and EEO) and CO_2_ (dosed) consumption. Moreover, direct CO_2_ utilization within a chain elongation metabolism cannot be excluded. It can be seen that EEO increased in phase III to 34.4% and 54%, for reactor 1 and reactor 2 respectively.

Once CO_2_ dosing was lowered in phase IV, EEO dropped to 0.3% and 3.0%, for reactor 1 and reactor 2 respectively. During this phase, ethanol was again no longer the limiting substrate (for reactor 1), as the ethanol broth concentrations remained between 700 and 1000 mCM. A peculiar observation is that the EEO in phase I seemed to be largely negative: −42.1% (reactor 1) and −38.4% (reactor 2). To calculate EEO, it is assumed that for each five chain elongation events, one ethanol is oxidized towards acetate. All additionally consumed ethanol is considered an “excess.” These results suggest the following: 1) a carbon/electron balance of 101% in phase I is slightly too high, as one would expect around 95% when taking into account biomass growth (Kottenhahn et al., 2021). Especially with the high ethanol concentrations used in this research, a small measurement deviation could affect such calculations tremendously. 2) The chain elongation microbiome utilizes a different stoichiometry with the applied substrate concentrations. Possibly, less ethanol is oxidized to acetate such that relatively more electrons and carbon atoms derived from ethanol are utilized within the reverse beta-oxidation. This would invalidate the assumption that for every 5 ethanol, 1 ethanol is oxidized to acetate and H_2_. However, hydrogen measurements in the headspace indicate at least some ethanol should be oxidized with H_2_ as a product.

Evidently the lowered CO_2_ loading rate decreased EEO from 34% to 53% in phase III to 0% and 3% in phase IV (See Supplementary Tables S2, S3) (Roghair et al., 2018a), for reactor 1 and 2 respectively. The large butyrate and ethanol load combined with acetate and CO_2_ limitation resulted in a stable chain elongation reactor that net consumed C_4_, while C_6_ was the main product and butanol and hexanol were produced from the corresponding carboxylates.

### Microbial community analysis: ingrowth and destruction of a *C. luticellarii* microbiome

**FIGURE 4:**
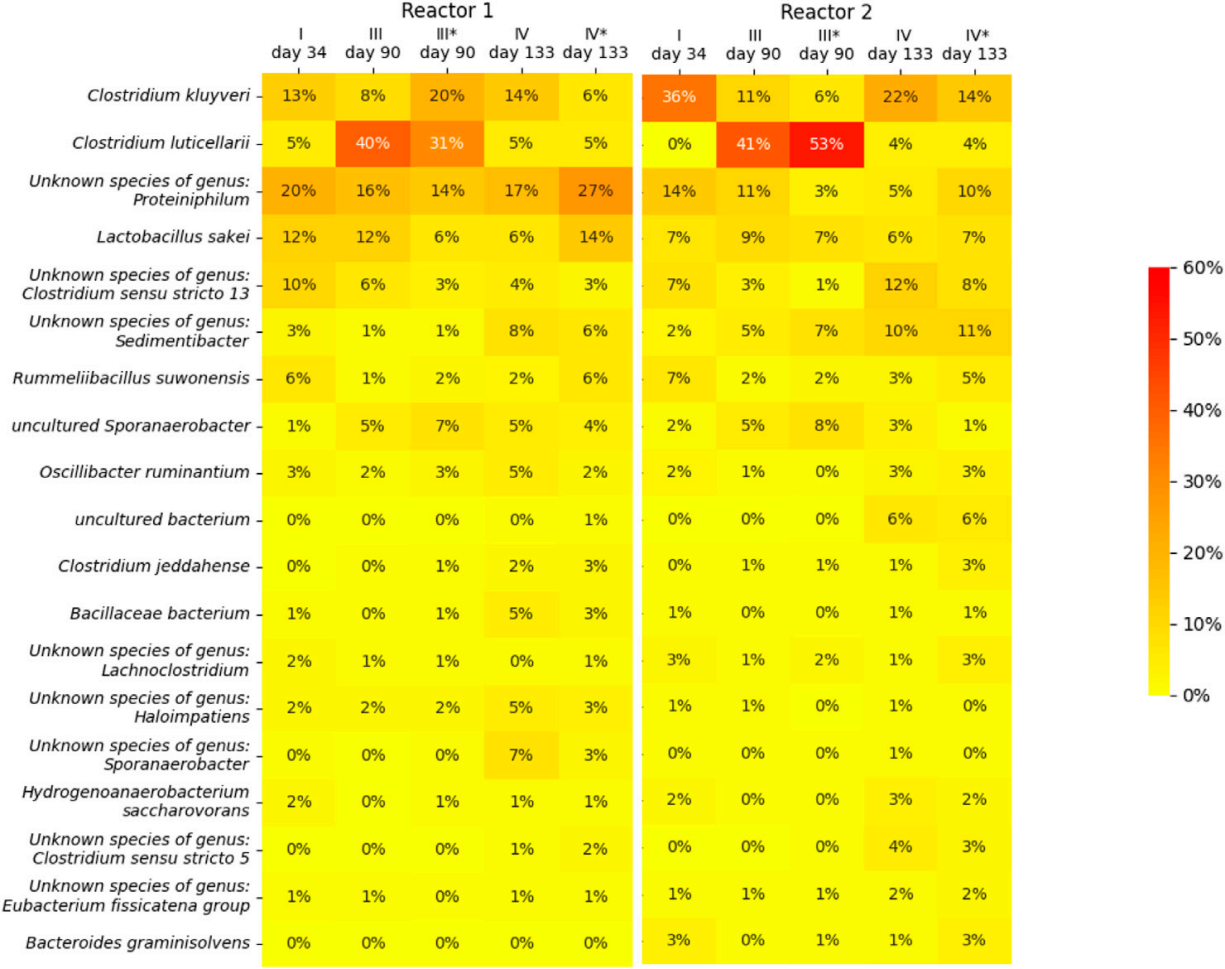
Heat map showing the microbial community composition in terms of relative abundance determined by 16s RNA analysis. An asterisk (*) indicates samples taken from the biofilm (phase III and phase IV), samples without asterisk were taken from the suspension (phase I, III and IV). Only microorganisms with a relative abundance of >3% are depicted.

For both reactors, the suspended biomass at the end of phase I, III and IV, as well as the biofilms at the end of phase III and IV, were sampled to perform a 16S rRNA gene amplicon microbial community analysis. A heat map of the microbial community analsys data is depicted in Figure 4, in which only microbial species are included of which the abundance in at least one of the samples is more than 3%. The heat map shows that species belonging to the *Clostridium* genera are the most dominant throughout the operation period of both reactor, together with an unknown species of the genus *Proteiniphilum* and *Lactobacillus sakei*. The changes in the CO_2_ caused a clear shift in the biomass species composition among the *Clostridium* genus. From phase I to phase III, this sheft led to the dominance of species related to *C. luticerllarii* when the carbon dioxie loading rate was at its peak (6 mL L^−1^ min^−1^). The abundance of *C. luticerllarii* diminished when the carbon dioxide loading rate was lowered again in phase IV (0.5 mL L^−1^ min^−1^).

**FIGURE 5:**
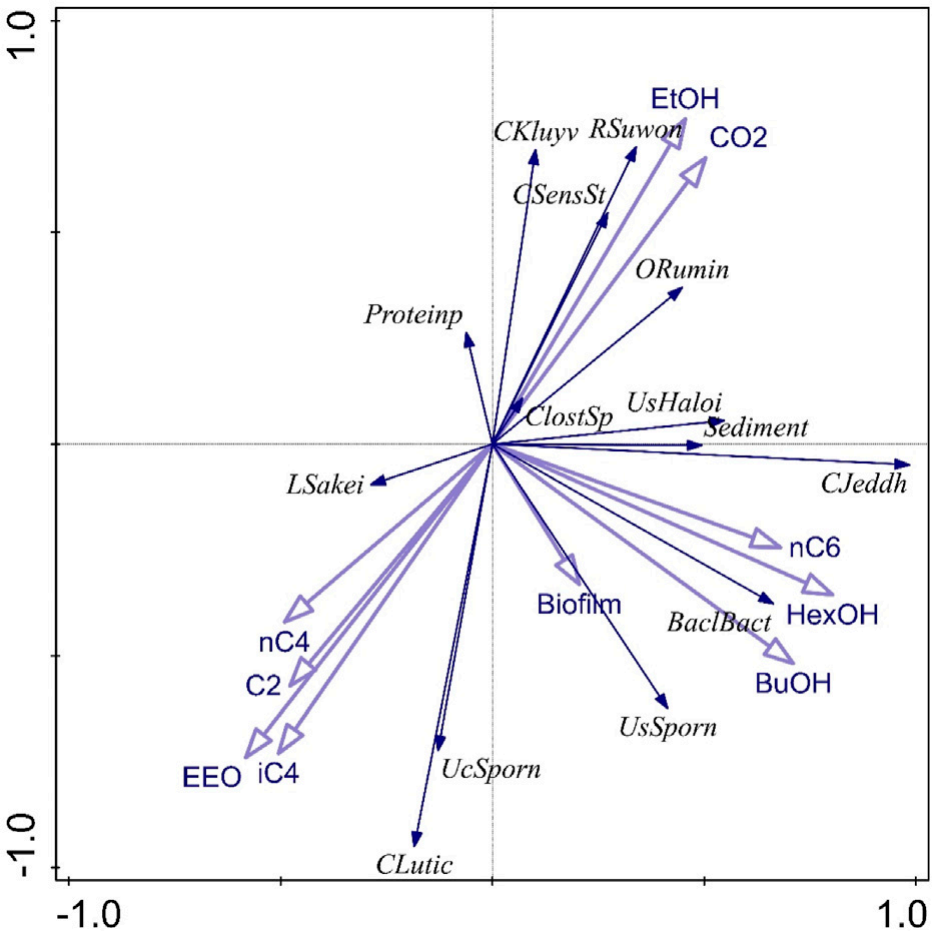
The figure shows an unconstrained redundancy triplot that was generated using the shown microbial composition as species data and the performance parameters in Supplementary Tables S2, S3 as environmental parameters.

The dominance of *C luticallarii* in phase III suggests that the species related to *C. luticellarii* in this study is largely responsible for the utilisation of CO_2_ and possibly also the (co)-utilization of ethanol to produce various carboxylates. *C. luticellarii* was found to utilize CO_2_ resulting in homoacetogenic activity and the formation of butyrate and isobutyrate (Petrognani et al., 2020). Moreover, this species is also described to perform chain elongation activity towards n-butyrate, i-butyrate, valerate and caproate (K. D. de Leeuw et al., 2020; De Smit et al., 2019), albeit with methanol as the electron donor. Interestingly, although previous research suggests *C luticellarii DSM 29 923* does not utilize ethanol (Petrognani et al., 2020), in this research *C luticellarii* shows the largest correlation with ethanol consumption (see Figure 5), together with an uncultured *Sporanaerobacter*. A genome analysis indicated that the *C. luticellarii* (taxid:1691940) genome in the NCBI database does contain genes for ethanol dehydrogenase, while also harboring the Wood-Ljungdahl pathway (K. de Leeuw, 2020). Given the increase in its relative abundance during phase III, it is likely that the *C. luticellarii* species in these bioreactors was largely responsible for ethanol oxidation coupled to CO_2_ reduction.

### Biofilm and suspended bacterial growth related to reactor performance

Reactor 1 and reactor 2 showed a peculiar difference in performance during phase III. The reactors definitely experienced different histories, which is reflected by the observed microbiomes. Although the suspension shows a similar microbiome profile, the biofilms have a distinctly different composition: reactor 1 biofilm contains a relative abundance of 20% for *C kluyervi* and 31% for *C luticellarii*, whereas reactor 2 biofilm contains a relative abundance of only 5% for *C kluyveri* compared to 53% for *C luticellarii*. Performance during phase III indicates that reactor 1 becomes a C_4_-producing system whereas reactor 2 maintains its C_6_ producing capacity alongside acetate formation.

Earlier research has shown that a high ethanol to acetate ratio stimulates longer chains, whereas a low ethanol to acetate ratio stimulates C_4_ production (Spirito et al., 2018). Hypothetically, the *C. kluyveri* experiences a lower ethanol to acetate ratio within the biofilm in reactor 1 compared to reactor 2 due to the ethanol oxidizing and homoacetogenic activity of *C. luticellarii*, leading to the two different observed reactor performances.

Although both reactors recovered to a similar performance profile in the final phase of the experiment, the resulting microbiomes did show large differences. For instance, in reactor 1 there was no clear return of *C. kluyveri*; in fact, its relative abundance dropped in the last phase. Instead, an unknown species of the genus *Proteiniphilum* and *Lactobacillus sakei* increased their relative abundance in this system. *Proteiniphilum* has been detected during fermentation of lignocellulosic ethanol to caproate and also in Chinese liquor clay pits (Wang et al., 2016; Liu et al., 2022), which suggest they may also play a role in chain elongation. A possible explanation for the abundance of *lactobacillus* could be attained to the abundance of amino acids derived from yeast extract in the medium, which can be also used for energy generation (Montanari et al., 2018). The different microbiome compositions within the reactors with similar performance suggest that the microbiome contains a certain functional redundancy; multiple possible microbiomes configurations can thrive in the environment while harbouring similar conversion capacities.

## Conclusion

We show that CO_2_ supply is a strong tool to control chain elongation reactor microbiomes and to stimulate solventogenesis by formation of higher alcohols or to stimulate homoacetogenesis to utilise CO_2_. Excessive ethanol consumption can, without CO_2_ supply, apparently be avoided entirely leading to selective n-caproate formation at 96 Cmol% selectivity and net butyrate consumption. At high CO_2_ loads homoacetogenesis and CO_2_ elongation are stimulated leading to a CO_2_ utilisation of up to 163 mCmol.L^−1^.d^−1^ (7.17 g.L^−1^.d^−1^) in this system. CO_2_ fixation seems to occur by among others a *C. luticellarii* related species. Hexanol formation was achieved with a productivity of 0.3 g L^−1^ day^−1^ (17.9 mCmol L^−1^ day^−1^) and a maximum concentration of 605 mg L^−1^ by operating the bioreactor with limited amounts of CO_2_ supply (0.5 NmL L^−1^ min^−1^) combined with a large overdose of ethanol and n-butyrate together with limiting acetate amounts.

## Data Availability

The microbial community data presented in the study are deposited in the European Nucleotide Archive (ENA) under accession number PRJEB74642. The bioreactor performance data are deposited in the 4TU database (https://data.4tu.nl) with DOI https://doi.org/10.4121/c76970fc-f617-46f0-9c83-f6f9e78f0a36.
