# Supplementary material for: CO2 supply is a powerful tool to control homoacetogenesis, chain elongation and solventogenesis in ethanol and carboxylate fed reactor microbiomes
Source: Front Bioeng Biotechnol. 2024 Apr 24;12:1329288. doi: 10.3389/fbioe.2024.1329288 (PMC11076876; doi:10.3389/fbioe.2024.1329288)
Supplement: Supplementary file 1 [file Presentation1.pdf]

Supplementary Material

CO_2 Supply is a Powerful Tool to Control Homoacetogenesis, Chain Elongation and Solventogenesis in Ethanol and Carboxylate Fed Reactor Microbiomes

Kasper D. de Leeuw[1,2], Marius J.W. van Willigen[1], Ton Vrauwdeunt[1], David P. P. T. B. Strik[1*]

[1]Environmental Technology, Wageningen University & Research, Wageningen, The Netherlands

[2]ChainCraft B.V., Amsterdam, The Netherlands

*** Correspondence:**
David P. P. T. B. Strik
david.strik@wur.nl

TABLE S.1. Overview over the different phases in the chemostat reactors. The first five days the reactors were in batch made; on the fifth day the first phase began. The duration of each phase, influent of carbon sources, CO_2 supply, HRT, pH and temperature are listed. Values given are the averages over the whole phase for each parameter with confidence interval (alpha 0.01). Besides the initial batch phase, both reactors experienced the following disturbances: on day 64 (power outage, 2 hours, temperature drop to 32.8ºC) and on day 106 to day 108 (2 days, feed pump off), leading to a short batch periods. Moreover, reactor 2 was also in batch mode from day 11-15 due to tube leakages.

	Phase 1		Phase 2		Phase 3		Phase 4	
CO2 supply (NmL L^{-1} min^{-1})	0.0		2.0		6.0		0.5	
Phase period (days)	5 - 34		34 - 57		57 - 91		91 - 133	
Acetate inlet (mM C)	50		50		50		50	
Butyrate inlet (mM C)	600		600		600		600	
Ethanol inlet (mM C)	1200		1200		1200		1200	
Reactor 1 operating conditions								
HRT (h)	46.9	± 7.6	41.9	± 5.8	40.2	± 9.1	42.8	± 1.0
Reactor temperature (°C)	35.3	± 0.1	35.2	± 0.1	35.1	± 0.1	35.1	± 0.0
pH	6.50	± 0.05	6.39	± 0.30	6.47	± 0.07	6.46	± 0.07
Carbon balance (%)	99%	± 3.6	94%	± 5.1	95%	± 7.9	100%	± 5.8
Electron balance (%)	100%	± 3.6	95%	± 6.2	96%	± 5.3	99%	± 6.4
Reactor 2 operating conditions								
HRT (h)	53.6	± 8.4	42.1	± 1.6	40.1	± 5.9	42.4	± 0.7
Reactor temperature (°C)	34.9	± 0.0	34.9	± 0.0	34.9	± 0.1	34.9	± 0.0
pH	6.7	± 0.2	6.6	± 0.1	6.5	± 0.0	6.5	± 0.0
Carbon balance (%)	100	± 1.8	96	± 7.2	94	± 4.2	96	± 2.1
Electron balance (%)	99	± 2.8	95	± 8.6	92	± 4.8	94	± 3.2

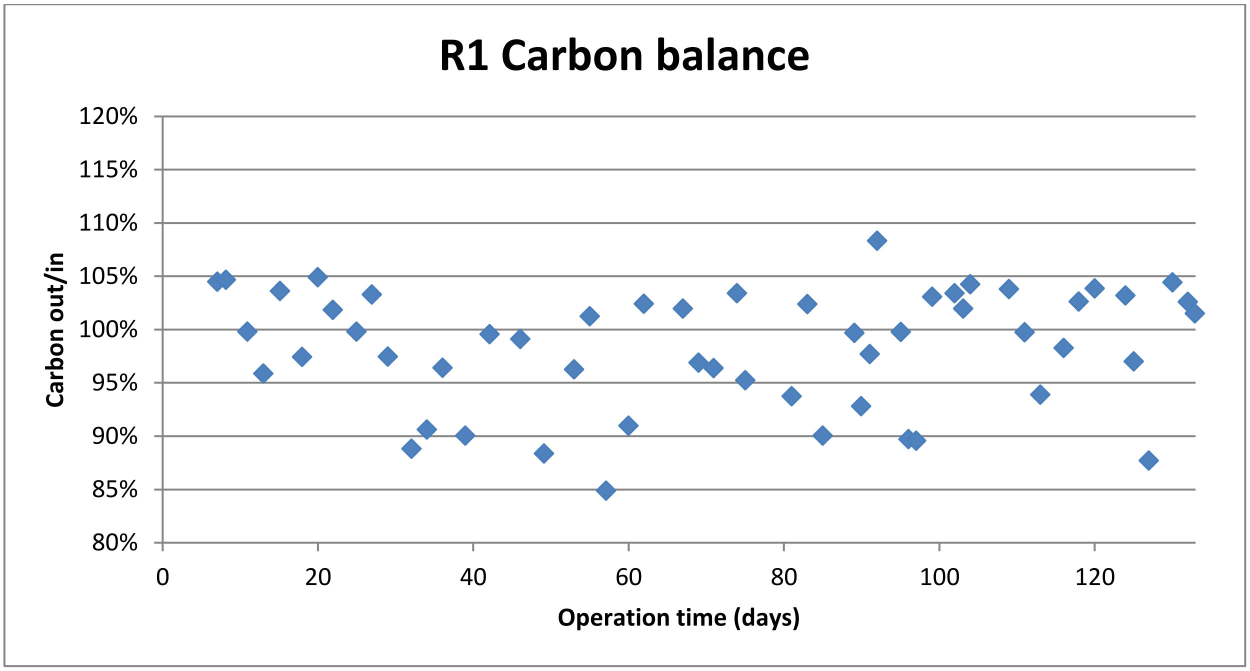

Figure S.1. Reactor 1 calculated carbon balance.

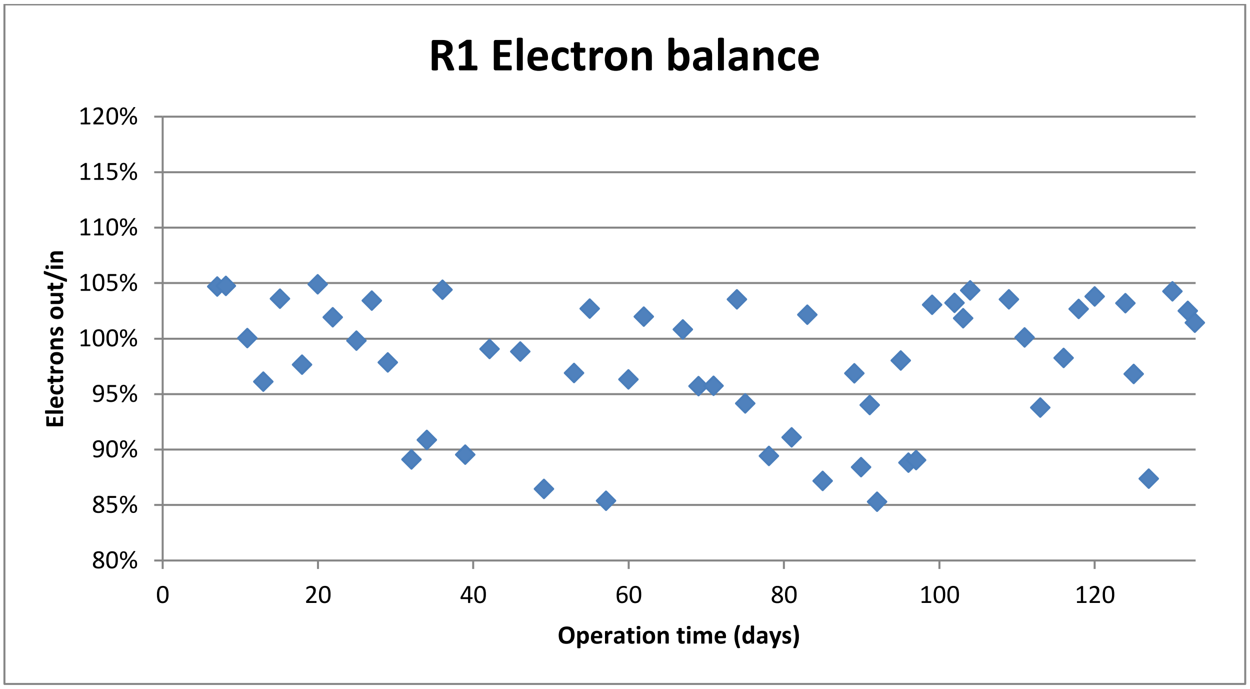

Figure S.2. Reactor 1 calculated electron balance.

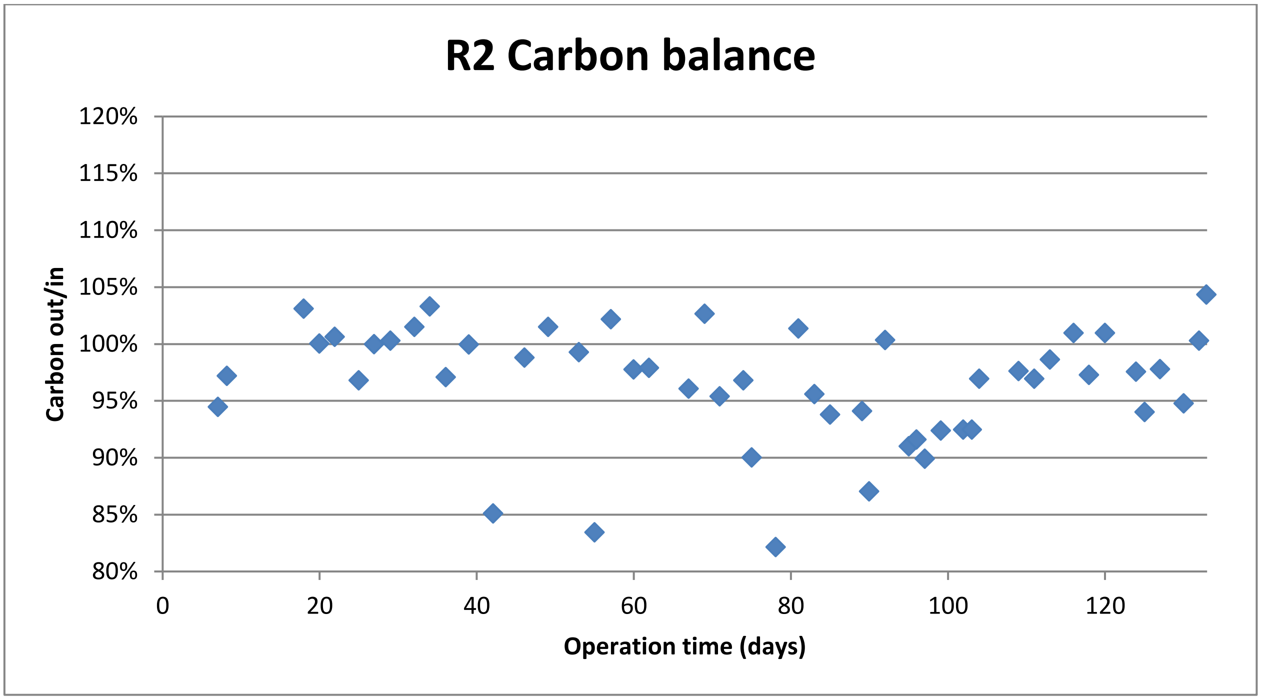

Figure S.3. Reactor 2 calculated carbon balance.

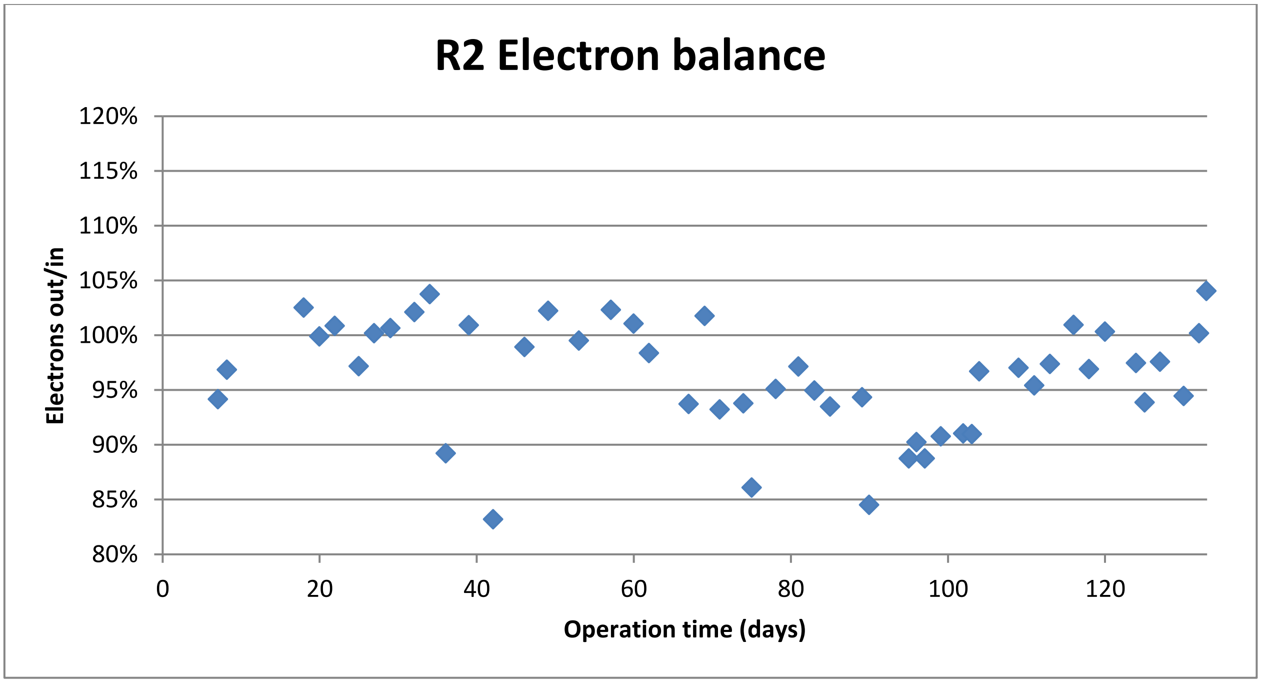

Figure S.4. Reactor 2 calculated electron balance.

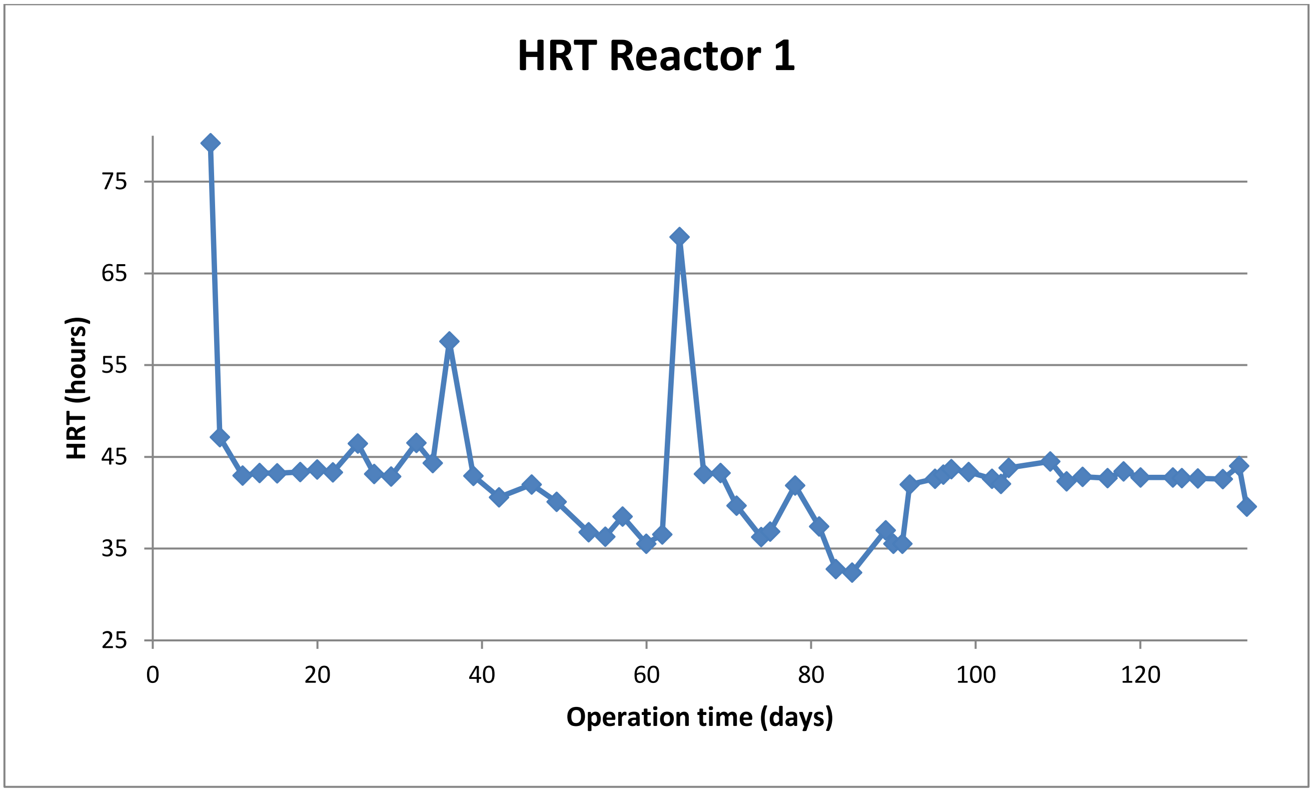

Figure S.5. Reactor 1 hydraulic retention time (HRT)

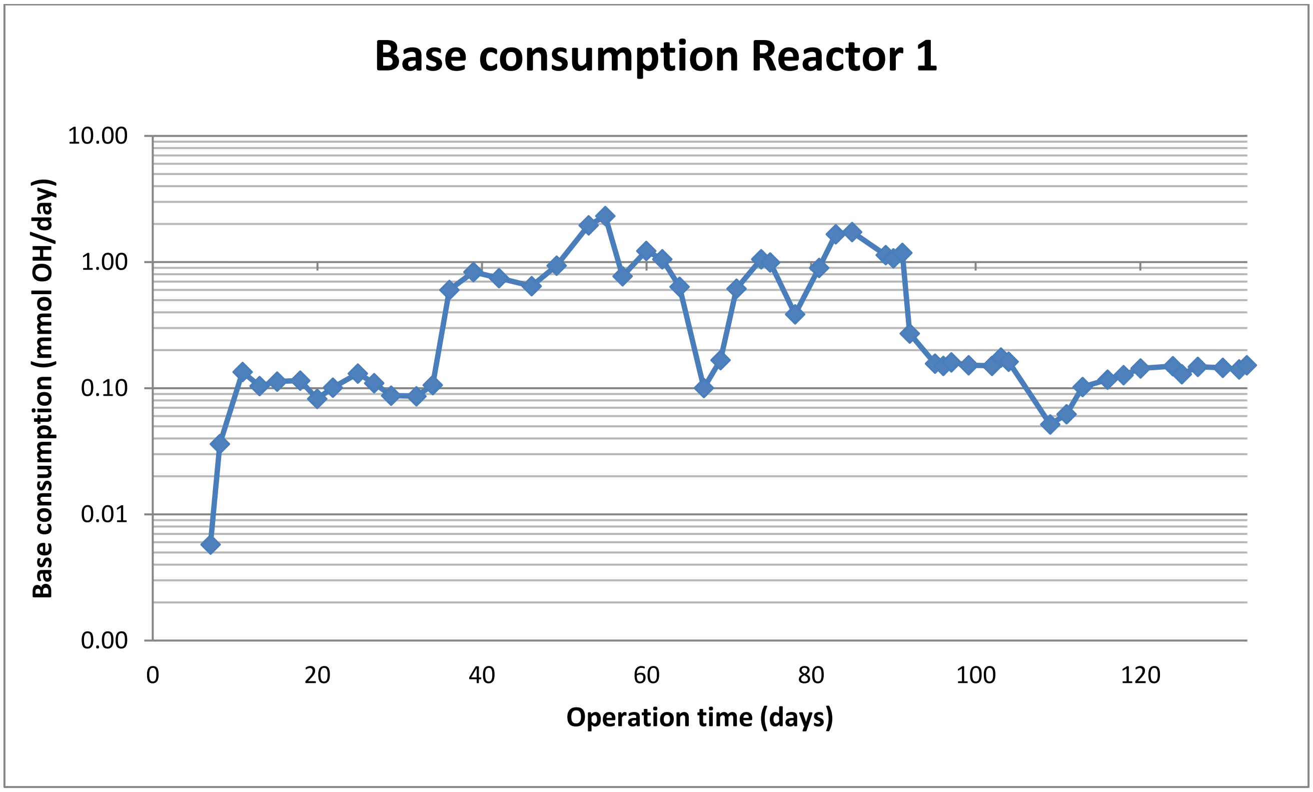

Figure S.6. Reactor 1 base consumption.

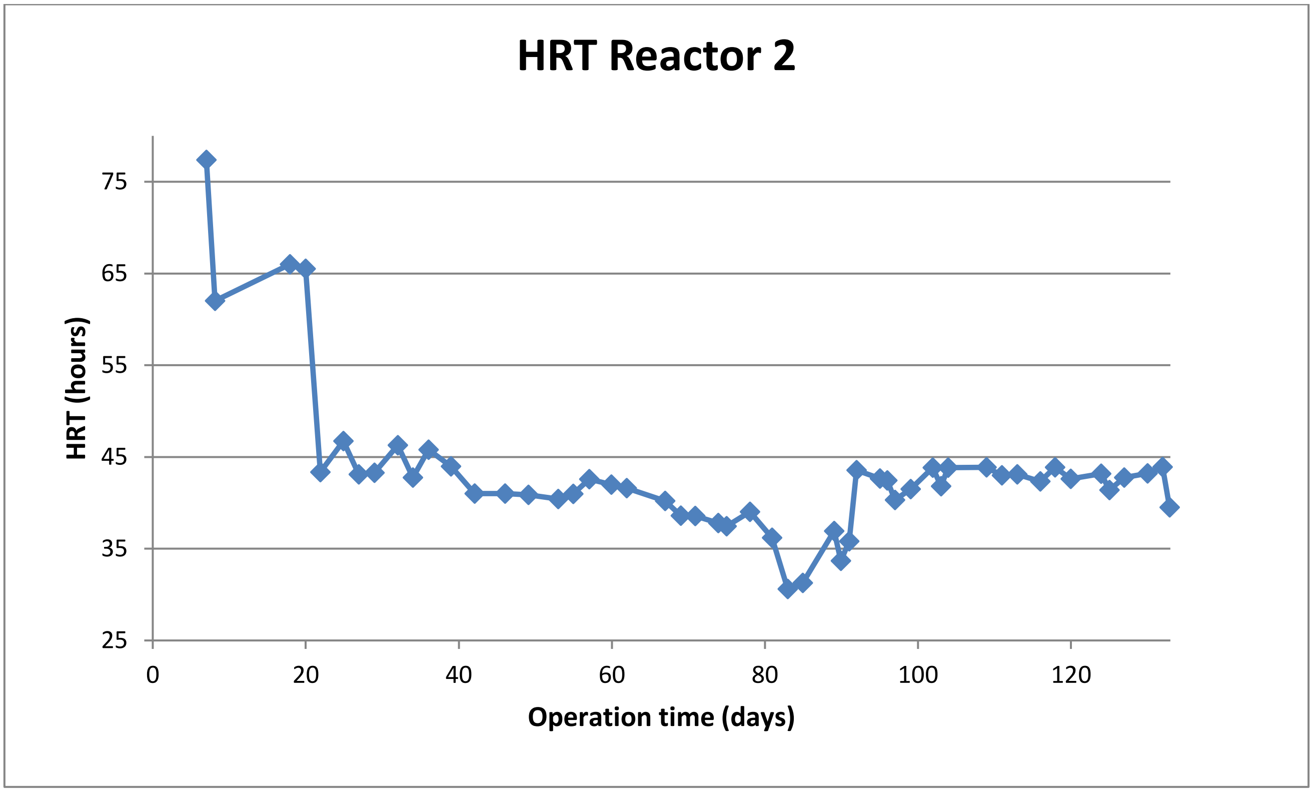

Figure S.7. Reactor 2 hydraulic retention time (HRT).

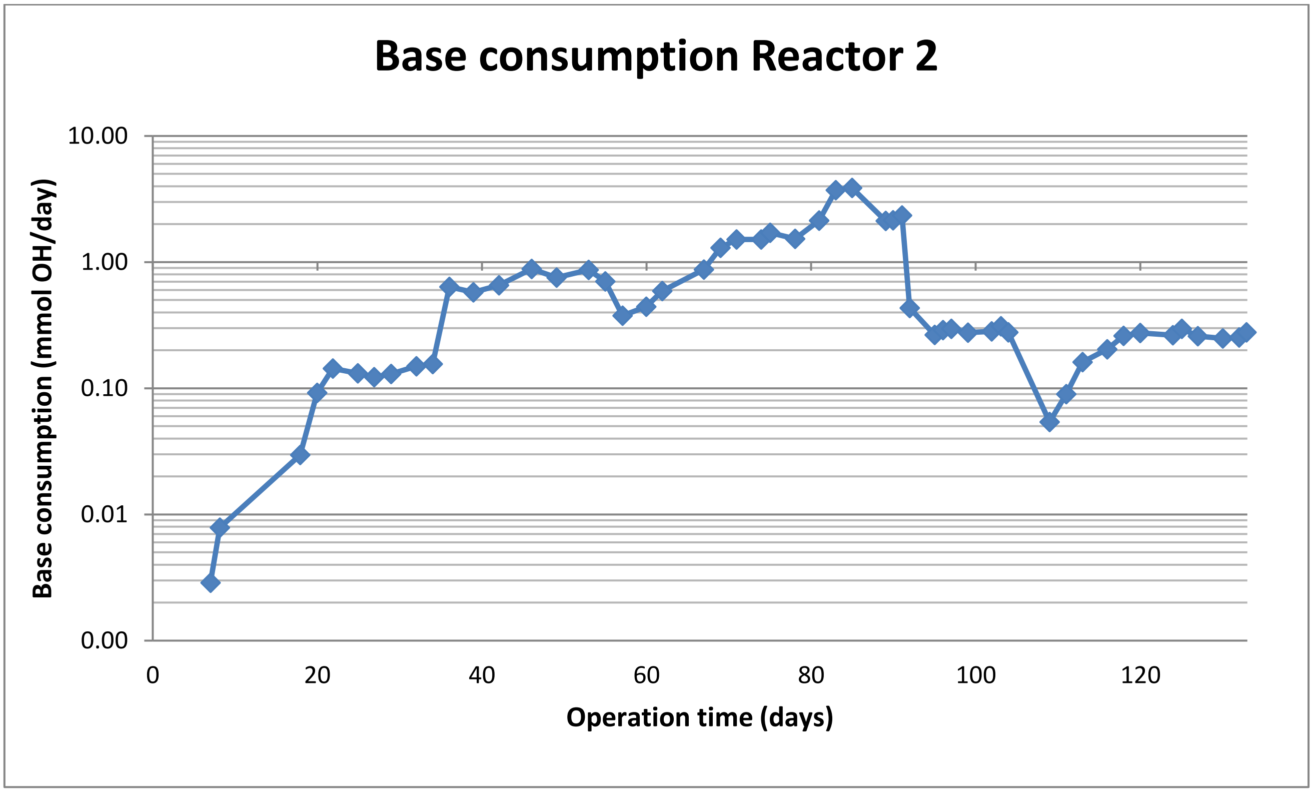

Figure S.8. Reactor 2 base consumption.

TABLE S.2. Overview of the steady state productivities during the four phases

Reactor 1 performance								
Phase	I		II		III		IV	
Steady state period (days)	no		no		no		124-133	
Averages period (days)	11-27		53-57		81-91		124-133	
Sample size of average and confidence interval	8		4		6		6	
**Volumetric C molar productivities (mCmol L-1 day-1)**								
C2	-14.3	± 1.4	135.0	± 100.1	79.3	± 46.9	-11.8	± 7.3
C3	0.0	± 0.0	0.2	± 0.5	0.5	± 0.4	0.0	± 0.0
i-C4	0.1	± 0.2	48.1	± 39.9	34.4	± 18.0	7.4	± 0.5
n-C4	-68.9	± 13.1	205.3	± 120.2	305.8	± 147.9	-27.3	± 20.9
i-C5	0.7	± 0.1	1.8	± 0.3	2.2	± 1.1	1.7	± 0.1
n-C5	0.0	± 0.0	0.2	± 0.3	0.0	± 0.0	0.0	± 0.0
i-C6	0.0	± 0.0	0.0	± 0.0	0.1	± 0.1	0.0	± 0.0
n-C6	218.8	± 10.7	50.5	± 42.5	170.9	± 82.0	298.6	± 24.0
n-C7	0.0	± 0.0	0.0	± 0.0	0.0	± 0.0	2.4	± 1.8
n-C8	2.8	± 0.3	0.2	± 0.2	0.0	± 0.0	3.2	± 0.6
Ethanol	-97.0	± 9.9	-388.3	± 126.6	-637.7	± 20.8	-244.6	± 27.2
Butanol	2.0	± 0.2	15.5	± 5.0	3.1	± 2.4	7.6	± 1.2
Hexanol	1.2	± 0.2	4.8	± 3.5	1.3	± 1.1	11.7	± 1.0
CO2	1.2	± 0.3	-12.0	± 8.7	-88.0	± 40.0	-14.4	± 0.6
Yeast extract*	-37.7	± 0.8	-37.0	± 1.6	-38.3	± 0.8	-38.2	± 1.3
EEO	-42%		12%		34%		0%	
**Concentrations (mM)**								
C2	11.7	± 1.3	125.1	± 74.3	86.1	± 17.6	13.9	± 6.5
C3	0.0	± 0.0	0.1	± 0.2	0.2	± 0.2	0.0	± 0.0
i-C4	0.0	± 0.1	18.9	± 16.4	15.5	± 3.0	3.3	± 0.3
n-C4	117.0	± 6.2	205.7	± 49.4	259.6	± 24.5	134.0	± 9.2
i-C5	0.2	± 0.0	0.6	± 0.1	0.8	± 0.1	0.6	± 0.1
n-C5	0.0	± 0.0	0.1	± 0.1	0.0	± 0.0	0.0	± 0.0
i-C6	0.0	± 0.0	0.0	± 0.0	0.0	± 0.0	0.0	± 0.0
n-C6	66.4	± 3.8	12.9	± 10.8	49.7	± 4.7	87.8	± 6.6
n-C7	0.0	± 0.0	0.0	± 0.0	0.0	± 0.0	0.0	± 0.0
n-C8	0.6	± 0.1	0.0	± 0.0	0.0	± 0.0	0.7	± 0.1
Ethanol	505.1	± 10.7	195.4	± 95.7	18.3	± 13.2	368.0	± 24.7
Butanol	0.9	± 0.1	6.0	± 1.9	1.1	± 0.9	3.3	± 0.5
Hexanol	0.4	± 0.1	1.2	± 0.9	0.3	± 0.3	3.4	± 0.2
**Headspace during period [%]**								
H2	78.4	± 7.6	0.3	± 0.3	0.1	± 0.0	0.9	± 0.4
O2	1.6	± 2.4	9.7	± 4.6	0.4	± 0.1	0.3	± 0.0
N2	18.0	± 9.2	53.0	± 34.6	1.1	± 0.3	95.2	± 0.7
CO2	2.2	± 0.7	29.3	± 43.6	98.4	± 3.9	3.8	± 0.2

* For the calculations it was assumed that all fed yeast extract was consumed.

TABLE S.3. Overview of the steady state productivities during the four phases

Reactor 2 performance								
Phase	I		II		III		IV	
Steady state period (days)	no		no		no		124-133	
Averages period (days)	27-34		53-57		83-91		124-133	
Sample size of average and confidence interval	4		3		5		6	
**Volumetric C molar productivities (mCmol L-1 day-1)**								
C2	-15.7	± 3.9	-4.1	± 4.5	297.8	± 41.9	2.0	± 5.1
C3	0.3	± 0.2	0.0	± 0.0	0.0	± 0.0	0.0	± 0.0
i-C4	0.3	± 0.6	2.2	± 0.4	26.3	± 6.6	7.2	± 0.4
n-C4	-71.3	± 6.9	-61.2	± 5.6	-21.0	± 55.5	-93.0	± 9.4
i-C5	0.3	± 0.4	1.3	± 0.2	2.3	± 0.3	1.6	± 0.2
n-C5	0.1	± 0.3	0.5	± 0.0	0.0	± 0.0	0.0	± 0.0
i-C6	0.0	± 0.0	0.0	± 0.0	1.0	± 0.3	0.0	± 0.0
n-C6	241.0	± 21.3	343.6	± 20.3	318.3	± 64.4	328.2	± 30.3
n-C7	0.0	± 0.0	0.0	± 0.0	0.0	± 0.0	0.0	± 0.0
n-C8	2.7	± 1.5	4.2	± 1.6	3.6	± 2.3	0.8	± 1.8
Ethanol	-111.2	± 7.4	-313.5	± 162.8	-590.4	± 44.2	-234.5	± 21.8
Butanol	1.8	± 0.4	5.8	± 1.1	5.5	± 1.0	5.9	± 0.9
Hexanol	1.0	± 0.7	10.9	± 1.2	10.2	± 2.6	14.4	± 0.9
CO2	1.5	± 0.1	-9.6	± 4.3	-163.4	± 14.0	-15.9	± 0.4
Yeast extract	-37.4	± 1.6	-37.7	± 0.4	-39.0	± 1.9	-38.5	± 1.7
EEO	-38%		14%		54%		3%	
**Concentrations (mM)**								
C2	10.3	± 3.7	20.0	± 0.2	226.8	± 11.9	26.1	± 4.5
C3	0.2	± 0.1	0.0	± 5.4	0.0	± 0.0	0.0	± 0.0
i-C4	0.1	± 0.3	1.0	± 0.0	9.1	± 1.6	3.2	± 0.2
n-C4	115.4	± 3.2	114.7	± 0.0	110.1	± 14.6	105.6	± 4.9
i-C5	0.1	± 0.2	0.5	± 0.0	0.7	± 0.1	0.5	± 0.0
n-C5	0.0	± 0.1	0.2	± 4.1	0.0	± 0.0	0.0	± 0.0
i-C6	0.0	± 0.0	0.0	± 0.0	0.2	± 0.1	0.0	± 0.0
n-C6	73.4	± 6.6	98.5	± 0.4	74.1	± 15.1	95.9	± 6.2
n-C7	0.0	± 0.0	0.0	± 0.0	0.0	± 0.0	0.0	± 0.0
n-C8	0.6	± 0.3	0.9	± 0.0	0.6	± 0.4	0.9	± 0.1
Ethanol	490.2	± 7.0	294.9	± 142.0	61.0	± 26.7	378.9	± 20.6
Butanol	0.8	± 0.2	2.5	± 0.4	1.9	± 0.4	2.6	± 0.3
Hexanol	0.3	± 0.2	3.1	± 0.0	2.4	± 0.7	4.2	± 0.2
**Headspace during period (%)**								
H2	82.2	± 7.7	11.3	± 2.1	0.4	± 0.1	1.0	± 0.6
O2	0.4	± 0.0	0.6	± 0.1	0.4	± 0.1	0.3	± 0.0
N2	6.9	± 2.8	2.0	± 0.2	1.3	± 0.6	94.9	± 0.8
CO2	2.5	± 0.1	86.3	± 4.8	97.9	± 3.6	4.0	± 0.3

* For the calculations it was assumed that all fed east extract was consumed.

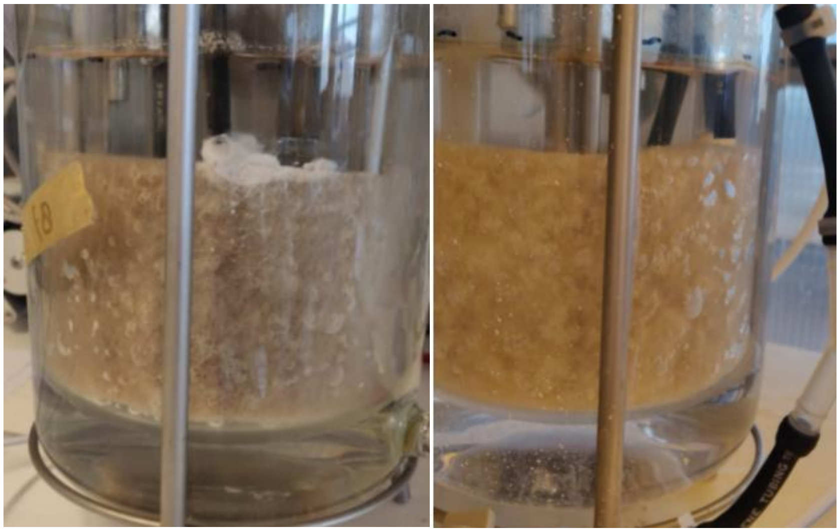

Figure S.9. Bubbles on the carrier material of reactor 1 (left) and reactor 2 (right). Pictures were taken on day 118.

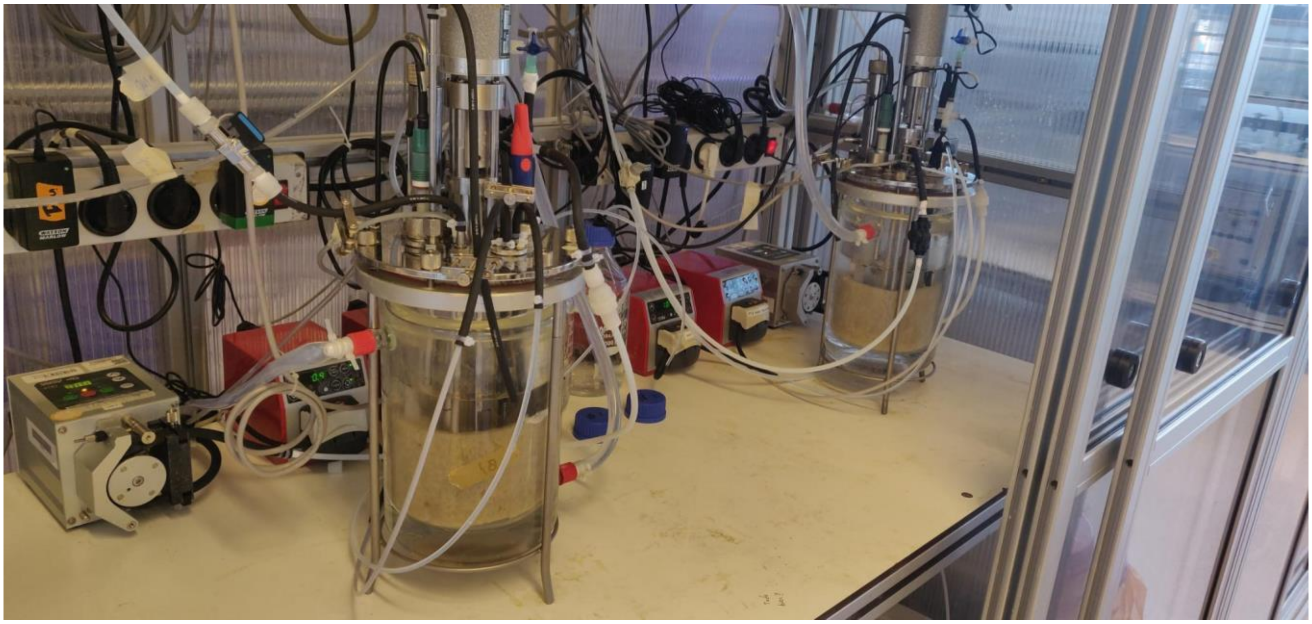

Figure S.10. The experimental set-up used with reactor 1 (left) and reactor 2 (right).

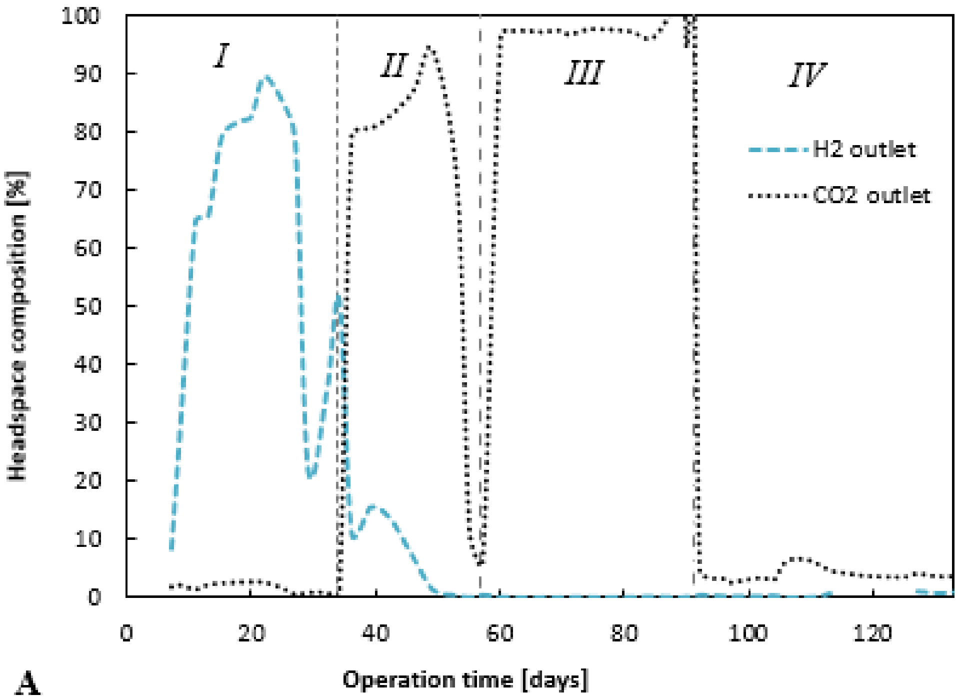

A

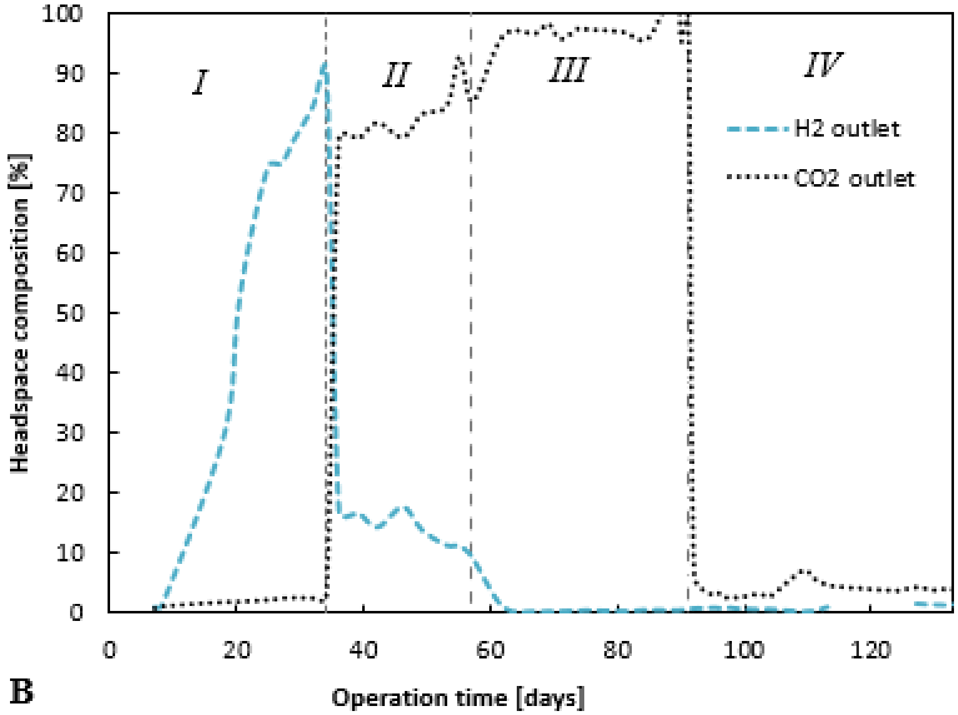

B

Figure S.11. The headspace composition (in percentages) for reactor 1 (A, upper) and reactor 2 (B, lower).
